# Unilateral Atraumatic Expulsion of an Ectopic Pregnancy in a Case of Bilateral Ectopic Pregnancy

**DOI:** 10.1155/2017/6391849

**Published:** 2017-09-27

**Authors:** Victoria Sampson, Oluremi Mogekwu, Ammar Ahmed, Farida Bano

**Affiliations:** Department of Obstetrics and Gynaecology, Queen's Hospital, Romford, UK

## Abstract

Ectopic pregnancy occurs in 1-2% of pregnancies. The fallopian tube is the most common site; however, bilateral tubal ectopic pregnancy is an extremely rare phenomenon, seen in approximately 1/200,000 pregnancies. It is usually the result of assisted reproductive techniques (ART). Ultrasound (USS) and serial beta-hCG levels have shown poor efficacy for accurate diagnosis. Laparoscopy is the diagnostic gold standard. The majority of cases are managed surgically with bilateral salpingectomy. A 26-year-old female presented to our early pregnancy unit with pain and vaginal bleeding at 5-week gestation after IVF. USS was inconclusive and her b-hCG levels rose with worsening pain; therefore, a decision was made for diagnostic laparoscopy. Although there was a clear right sided ectopic pregnancy, the left tube was swollen and therefore a methylene blue dye test was carried out to confirm blockage. Atraumatic milking, to expose the dye, expelled necrotic tissue which histology confirmed to be a second ectopic pregnancy. She made a good recovery with falling beta-hCG levels and left tubal preservation. As the use of ART increases, bilateral ectopic pregnancies will become more common. Novel and established techniques should be used to help confirm the diagnosis and assist in tubal preservation.

## 1. Introduction

Ectopic pregnancies (EPs) constitute 1-2% of all pregnancies [1] and are a leading cause of first-trimester maternal mortality [2]. Ectopic pregnancy describes implantation of a blastocyst outside of the uterine cavity. The fallopian tubes are the most common site, accounting for 95% of EPs [3]. Key risk factors include previous EP, known tubal damage, pelvic inflammatory disease, presence of an intrauterine device, smoking, assisted reproductive techniques (ART), and extremes of maternal age [2, 4].

Bilateral tubal ectopic pregnancies (BEPs) are a very rare form of ectopic pregnancy [5], and incidence has been reported as 1 in 725 to 1 in 1580 of ectopic pregnancies equating to approximately 1 in 200,000 pregnancies [6].

The majority of cases are diagnosed intraoperatively; traditional diagnostic methods such as serial beta-human chorionic gonadotrophin levels (beta-hCG) or transvaginal ultrasound have shown poor efficacy when applied to BEPs [5]. Management is typically with bilateral salpingectomy.

We present a case of bilateral ectopic pregnancy in which diagnosis was assisted by the use of methylene blue dye test. It was managed with unilateral salpingectomy and preservation of the remaining fallopian tube following atraumatic expulsion of the contralateral ectopic pregnancy during tubal insufflation.

## 2. Case Report

A 26-year-old Caucasian woman presented to our early pregnancy unit with five-week amenorrhea, sudden onset of abdominal pain, and vaginal bleeding for one week. On initial assessment, her vital signs were stable. She achieved conception via in vitro fertilization (IVF) on her fourth attempt; two blastocysts were implanted 36 days prior to presentation. She had one live child aged 4 years delivered vaginally and three early recurrent miscarriages. There was no further significant medical history.

Investigation with a transvaginal ultrasound scan (TVUS) showed an endometrial thickness of 2.5 mm with interrupted midline echo, and no free fluid was noted. A diagnosis of pregnancy of unknown location was made but a complete miscarriage was suspected. Subsequently, serum beta-hCG and serum progesterone levels were checked to correlate with the clinical picture. She was offered pain relief and she was advised of admission due to her pain, both of which she declined. The patient was advised to return in 48 hours for repeat blood tests. A normal rise in serum beta-hCG levels from 618 IU/L to 1290 IU/L over a 48-hour interval was noted.

A repeat TVUS was carried out at this stage. This showed an endometrial thickness of 4.7 mm, and a likely ectopic pregnancy on the left measuring 16 mm × 19 mm was noted. The patient was informed about the findings and was counseled accordingly. Risks and benefits of laparoscopy ± salpingectomy versus methotrexate were discussed with the patient and she decided to opt for surgery.

**Figure 1:**
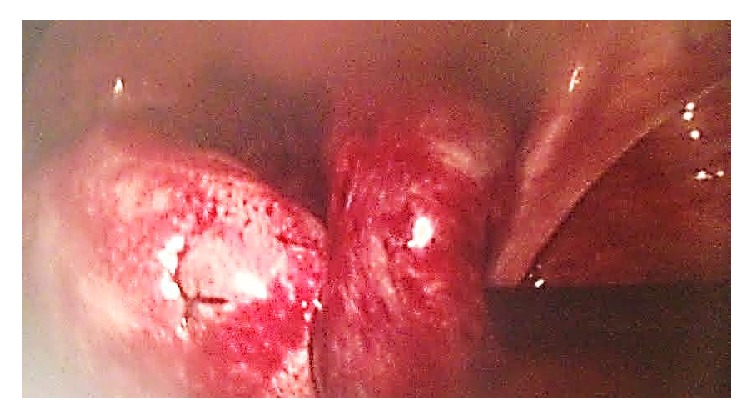
Left hematosalpinx.

**Figure 2:**
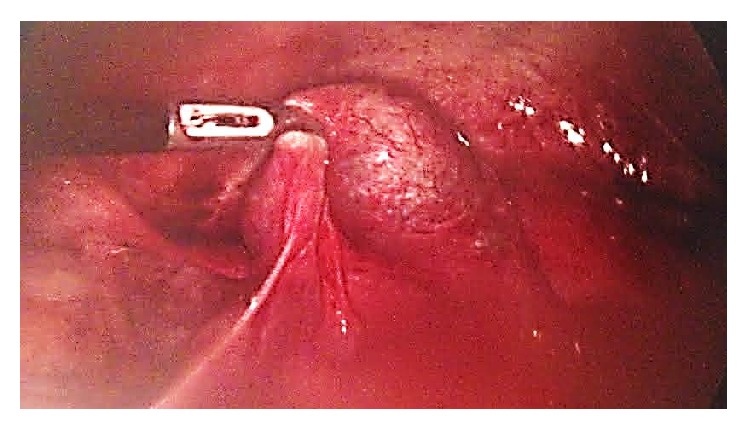
Right tubal ectopic pregnancy.

**Figure 3:**
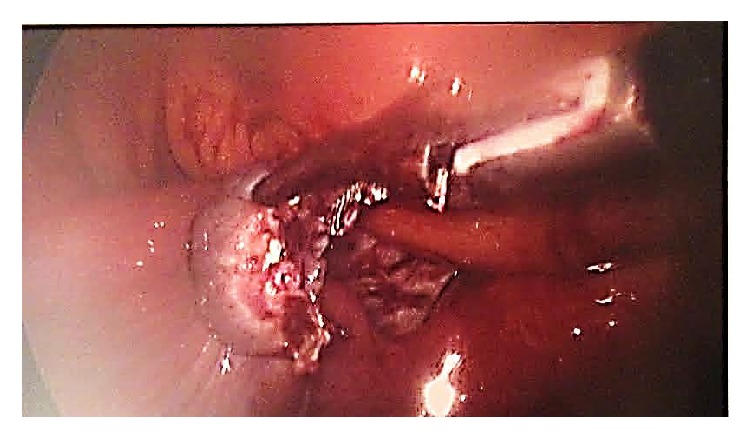
Right fallopian tube after salpingectomy.

A diagnostic laparoscopy was carried out; there was a mild hemoperitoneum. The left fallopian tube appeared edematous and dilated indicating a possible hematosalpinx or ectopic pregnancy (Figure 1). Simultaneously, there was a definite right ectopic pregnancy (Figure 2), and as a result a right salpingectomy was carried out, without any complications (Figure 3).

**Figure 4:**
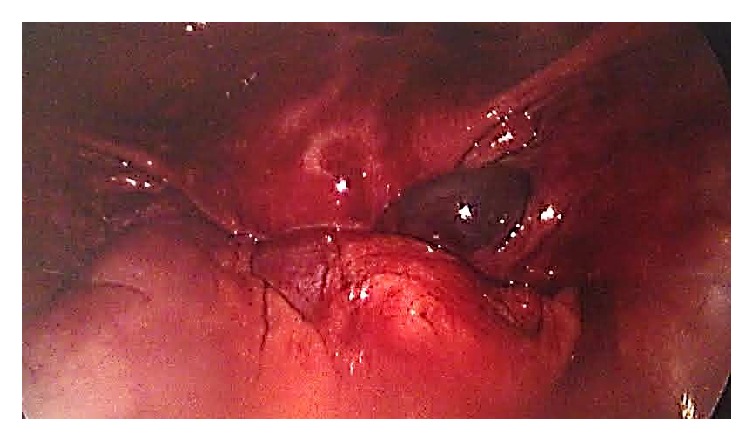
Laparoscopic picture of the tissue from the left fallopian tube.

Given the uncertainty regarding the left tube, a methylene blue dye test was carried out on the left tube and a small amount of blue spillage was noted. The left fallopian tube was then maneuvered with atraumatic forceps, in a milking motion, until a necrotic-looking tissue was released at the level of the left fimbria (Figure 4).

Good hemostasis was achieved and the total blood loss was estimated to be 250 ml. Both tissue samples were sent for histology. The patient had an uneventful recovery and was discharged home with the plan to return for follow-up in one weeks' time for repeat serum beta-hCG and ultrasound scan. Follow-up of this nature was planned due to the uncertainty of the content of the remaining tube; histology results would not be available for a minimum of two weeks. If there was a contralateral ectopic pregnancy, resolution of b-hCG would assist in excluding residual trophoblastic tissue. As this case had not been encountered previously, the repeat ultrasound was completed mainly for reassurance.

The following week, blood tests confirmed an optimal decline of beta-hCG levels and ultrasound scan was normal. Histology report confirmed the presence of chorionic villi and decidua in both tissue samples, confirming the diagnosis of bilateral ectopic pregnancy. The patient made an uneventful recovery.

## 3. Discussion

Any ectopic pregnancy is a potential medical emergency. Late or misdiagnosis can result in serious complications such as tubal rupture, hemorrhagic shock, and death [7]; yet, timely diagnosis, especially of bilateral tubal ectopic pregnancy, has proven to be particularly challenging.

Unlike unilateral ectopic pregnancies, measurement of serum beta-hCG levels for bilateral cases is neither a sensitive nor a reliable diagnostic marker given the presence of two pregnancies [8]. Furthermore, the efficacy of preoperative ultrasound in diagnosing BEPs is also poor with only a couple of successful cases known [9]; most cases identify one ectopic pregnancy or the patient presents in an unstable condition. Our case reiterates this difficulty with the initial ultrasound scan confirming an ectopic pregnancy on the left but was unable to visualize the ectopic pregnancy on the right, but intraoperatively the right sided ectopic pregnancy was much clearer. The current established method of diagnosing the second ectopic pregnancy is by direct inspection of the contralateral tube intraoperatively. Despite this, there have been cases of missed bilateral ectopic pregnancies resulting in a second emergency surgery following contralateral rupture [10].

Regarding unilateral EP, salpingectomy (tube removal) and salpingostomy (ectopic removal with tubal preservation) are the two surgical options. As tubal damage is the biggest risk factor for recurrence, salpingectomy is the preferred surgical management if the contralateral tube appears normal [11]. Salpingostomy can be considered if there are concerns about tubal factor infertility; it has been thought that salpingostomy was the preference over salpingectomy in order to preserve fertility [12]. Cheng et al. performed a meta-analysis comparing the fertility outcomes after salpingostomy versus salpingectomy; they included two randomized controlled trials and eight cohort studies. They found that the two RCTs did not indicate a significant difference between the two groups whereas the cohort studies suggest an increased intrauterine pregnancy (IUP) rate in the salpingostomy group. However, when excluding 2 of the cohort studies, they saw no significant difference between the IUP rates of the two treatment options [13].

BEPs pose management dilemmas as both tubes are damaged, resulting in a high risk of recurrence. In the literature, the majority of cases are managed with bilateral salpingectomy [14, 15]. There have been cases of successful conservative surgery [6] but also cases associated with persistent symptoms requiring further surgery or treatment with methotrexate [16, 17].

In our case, the patient was already undergoing IVF treatment, bypassing the need for tubal preservation. Women undergoing fertility treatment with tubal disease secondary to hydrosalpinx or tubal factor infertility have been shown to have a lower success rate of IVF compared to other causes of infertility [18]. This has led to the thought that sapling fluid can be embryotoxic by preventing implantation or being detrimental to the embryo development [18, 19]. A Cochrane review including 9 studies looked at the effect of surgical intervention in tubal factor infertility; the review found strong evidence for salpingectomy or tubal occlusion prior to IVF treatment in the case of hydrosalpinx [19], which is therefore a recommended practice [20]. Therefore, an argument could be made to offer all women with BEPs, secondary to IVF, bilateral salpingectomies in the hope of improving future outcomes. For our patient, the low index of suspicion for BEPs meant the option of bilateral salpingectomy was not considered especially as the diagnosis was not confirmed until the histology result was available.

Assisted reproductive techniques are a known risk factor for ectopic pregnancies [21]; the risk varies according to the technique employed with intrafallopian transfers conferring the highest risk [22, 23], a technique which has fallen out of practice. Regarding intrauterine transfers, midfundal techniques reduced the risk of ectopic pregnancy by 75% when compared to the deep fundal technique [22]. A large multicenter trial showed that the risk with ART is significantly higher if there is preexisting tubal factor infertility [24]. The rates of ectopic pregnancy secondary to ART have decreased over the past decade, likely secondary to improvements in techniques. Reduction in the number of embryos transferred has also contributed to the reduced risk [25]. Zhu et al. found that, of the 16 case reports of bilateral ectopic pregnancy since 2008, 43% were associated with assisted reproduction [26]. This figure is slightly less than previous literature reviews quoting 50% and 64%, respectively [27, 28]. Some cases thought to be spontaneous have revealed intraoperative signs of concealed ovarian induction [5]. Risk factors for spontaneous BEPs are similar to those for unilateral EPs and there are no established differences in their clinical presentation [5, 16].

As demonstrated, BEPs are difficult from a diagnostic and management perspective. In the absence of clinical guidance, new and innovative ideas are required to establish the best practice in these rare cases. Methylene blue tubal insufflation is commonly used at laparoscopy to identify tubal obstruction in cases of subfertility. Our case represents a novel approach in using methylene blue to assist the diagnosis of a contralateral ectopic pregnancy where intraoperative inspection was inconclusive.

An unexpected outcome was the atraumatic expulsion of the pregnancy resulting in the preservation of the tube. Complete removal of the ectopic pregnancy was confirmed by resolution of the b-hCG levels. For this to be considered a treatment option for other women, functionality of the tube must be established with a subsequent spontaneous intrauterine pregnancy. This must be weighed up against the risk of a recurrent ectopic pregnancy. Further cases are needed to ascertain trends.

In conclusion, as the use of assisted reproductive techniques increases, bilateral ectopic pregnancies will become more common. A high index of suspicion should be used in high risk cases with extra care taken to evaluate the contralateral tube. Both novel and established techniques should be used to ensure accurate diagnosis of which tubal insufflation may assist as not only diagnostic but also potential treatment option.
